# Ultrahigh Purcell factor in low-threshold nanolaser based on asymmetric hybrid plasmonic cavity

**DOI:** 10.1038/srep33063

**Published:** 2016-09-12

**Authors:** Wei Wei, Xin Yan, Xia Zhang

**Affiliations:** 1 Department of Physics, University College Cork, Western Road, Cork, Ireland; 2 Tyndall National Institute, Lee Maltings, Cork, Ireland; 3 State Key Laboratory of Information Photonics and Optical Communications, Beijing University of Posts and Telecommunications, P. O. Box 66, Beijing 100876, China

## Abstract

A low-threshold nanolaser with all three dimensions at the subwavelength scale is proposed and investigated. The nanolaser is constructed based on an asymmetric hybrid plasmonic F-P cavity with Ag-coated end facets. Lasing characteristics are calculated using finite element method at the wavelength of 1550 nm. The results show that owing to the low modal loss, large modal confinement factor of the asymmetric plasmonic cavity structure, in conjunction with the high reflectivity of the Ag reflectors, a minimum threshold gain of 240 cm^−1^ is predicted. Furthermore, the Purcell factor as large as 2518 is obtained with optimized structure parameters to enhance rates of spontaneous and stimulated emission.

The performance, speed and ease-of-use of semiconductor devices, circuits and components are dependent on their miniaturization and integration into external devices. However, the integration of modern electronic devices for information processing is rapidly approaching their fundamental speed and bandwidth limitations, which impedes applications in the areas of information and communications^1^. Optical signals, which do not suffer capacitive loading effects as in electronics, are promising to replace electronic signals as information carriers. Like most other photonic components, the minimum size for a laser is ultimately governed by the wavelength. Current semiconductor lasers composed of micro-scale resonating cavities require large dimensions compared to the wavelength, such as photonic crystal^2^,^3^,^4^ and distributed Bragg reflectors^5^,^6^,^7^. When all three dimensions of a conventional semiconductor laser are scaled down towards the wavelength, there are adverse effects in the lasing process which would hinder the lasing, such as shortened roundtrip path in the gain medium and decreased lateral field confinement. By allowing the laser size to increase in one or two dimensions, it is possible to reduce the physical size of the laser in the remaining dimension(s) to values smaller than the wavelength. For example, semiconductor nanowire lasers shrink diameters below the wavelength, whereas to provide sufficient roundtrip gain, the lengths of nanowires are still too long compared to the wavelength^8^,^9^,^10^,^11^. The challenge of this respect is simultaneous reduction of the cavity size in all three dimensions, while still satisfying the requirements for lasing action. One approach to solve this issue is to incorporate metals into the structure of dielectric cavities, because metals can suppress leaky optical modes and effectively isolate them from their neighboring environment^12^,^13^,^14^,^15^.

In this paper, we propose low-threshold nanolaser based on asymmetric hybrid plasmonic structures and Ag-coated end facets to form Fabry-Perot (F-P) cavity. The asymmetric hybrid plasmonic F-P cavity combines the advantages of hybrid^16^,^17^,^18^ and symmetric^19^,^20^,^21^ plasmonic modes supporting long-range propagation under nanoscale modal confinement. Taking advantage of the strong optical confinement of the asymmetric hybrid plasmonic waveguide and the high reflectivity of the Ag reflecting mirrors, all three dimensions of the nanolaser reduces below the free-space wavelength. Lasing characteristics of nanolaser were simulated using finite element method (FEM) at the wavelength of 1550 nm to target applications in telecommunications. Owing to the low modal loss and nanoscale modal area of the asymmetric hybrid plasmonic cavity, the nanolaser has a low threshold gain, along with an ultrahigh Purcell factor highly enhancing rates of spontaneous and stimulated emission and makes lasing easier. This nanolaser may find potential applications in on-chip short-distance communications, photonic integrated circuits and optical signal process.

The schematic diagram of the proposed nanolaser is demonstrated in Fig. 1. The nanolaser is on a silica substrate and surrounded by air. The height and width of the nanolaser are *H* = 300 nm and *W* = 200 nm. The Ag nanowire, of diameter *D* is embedded in the low-index silica region, locates 30 nm underneath the center of the nanolaser. The high-index region surrounding the low-index region is the gain medium made of InGaAsP. *W*_*l*_ and *H*_*g*_ denote the width of the low-index region and the height of the low-index gap between Ag nanowire and gain medium. *W*_*l*_ is fixed at 100 nm. The two Ag-coated end facets act as reflecting mirrors forming a F-P cavity for lightwaves oscillating in the asymmetric hybrid plasmonic cavity. The length of the F-P cavity is denoted by *L*. It is important to indicate that although direct growth of III-V semiconductor on Ag nanowire is not realistic, in the proposed nanolaser, the Ag nanowire is surrounded by a silica strip following InGaAsP, it is realistic to grow InGaAsP enclosing the silica strip due to the small diameter of silica strip. So the silica strip together with the Ag nanowire will be surrounded by InGaAsP during the growth process. The similar case of growing GaAs to coalesce above silica trenches has been experimentally verified in ref. 22. Thus, it is possible to realize the growth of InGaAsP coalescing and enclosing the silica strip with the Ag nanowire embedded in it.

## Results

**Figure 2:**
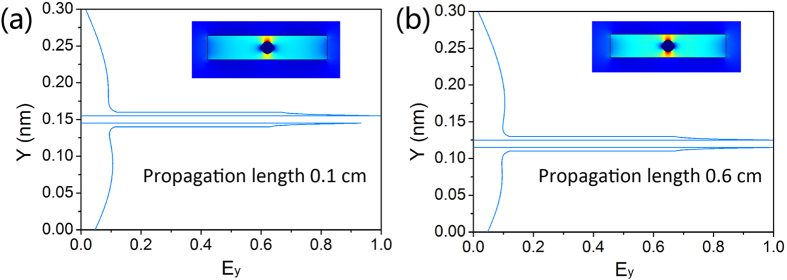
Modal profiles and propagation lengths of nanolaser. (**a**) Ag nanowire in the center of nanolaser and (**b**) 30 nm below the center of nanolaser.

In the structure of nanolaser, the Ag nanowire is embedded in the low-index silica region, locates 30 nm underneath the center of the nanolaser. This deviation of Ag nanowire away from the center of InGaAsP forms the asymmetric plasmonic structure, which can symmetrize the broken modal profiles. Modal profiles and propagation length of Ag nanowire in the center of the nanolaser and 30 nm below the center of the nanolaser are calculated using FEM at 1550 nm and shown in Fig. 2. The refractive index of Ag is taken from ref. 23. Since the symmetric hybrid plasmonic mode is quasi-TM in nature, the transverse and vertical components of electric field both exist, and the transverse field component *E*_*y*_ dominates. So, we demonstrate the *E*_*y*_ profiles of the symmetric hybrid plasmonic mode here. As shown in Fig. 2, the asymmetric structure symmetrizes the broken symmetric hybrid plasmonic modal profile induced by substrate, increases the propagation length, and decreases the propagation loss of the nanolaser under almost same mode confinement. The symmetric plasmonic mode in the asymmetric plasmonic structure has symmetric modal profile, which is beneficial to the lasing pattern.

**Figure 3:**
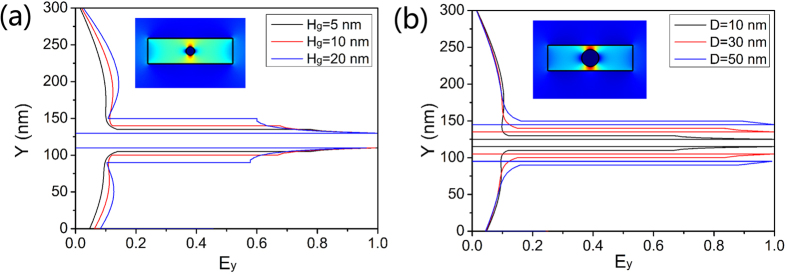
Magnitudes of *E*y along Y-axis. (**a**) *D* is fixed at 10 nm with different *H*_*g*_, (**b**) *H*_*g*_ is fixed at 5 nm with different *D*. Insets in (**a**,**b**) are modal profiles corresponding to *D* = 10 nm, *H*_*g*_ = 10 nm and *D* = 20 nm, *H*_*g*_ = 5 nm, respectively.

The diameter *D* of Ag nanowire and silica height *H*_*g*_ between the Ag nanowire and InGaAsP are two key factors to impact on the modal profiles of the hybrid plasmonic mode and further lasing properties of the nanolaser. The magnitudes of normalized *E*_*y*_ along the *Y*-axis for different *H*_*g*_ are depicted in Fig. 3(a). The electric field component *E*_*y*_ has symmetric distribution along the two vertical sides of the Ag nanowire, which is called symmetric hybrid plasmonic mode. The majority of *E*_*y*_ concentrates around the interface of Ag and SiO_2_ in the low-index region and attenuates rapidly away from the interface. When *H*_*g*_ is 5 nm, the hybrid plasmonic mode has the strongest confinement. The electric field are tightly confined around the surface of the Ag nanowire. This distributed width of electric field increases with increasing low-index height *H*_*g*_, indicating weakened coupling between plasmonic and dielectric modes. The magnitudes of normalized *E*_*y*_ along the *Y*-axis for different diameters of Ag nanowire *D* are depicted in Fig. 3(b). As the low-index height *H*_*g*_ is fixed at 5 nm, the widths of upper and under parts of the hybrid plasmonic mode in the low-index gap are almost the same for different *D*. Moreover, we find that as the diameter *D* becomes larger, *E*_*y*_ attenuates more slowly away from Ag nanowire and has a more homogeneous distribution in the low-index gap. So with the increased diameter of Ag nanowire, the coupling between plasmonic mode and dielectric mode becomes stronger. It is also worth mentioning that, small diameters of Ag nanowires ranging from 10 to 50 nm are realistic, which can be synthesized with high aspect-ratio (8–10 nm in diameter and length up to 10 μm) by the reduction of AgNO_3_ with Vitamin C in SDS/ethanol solution^24^.

**Figure 4:**
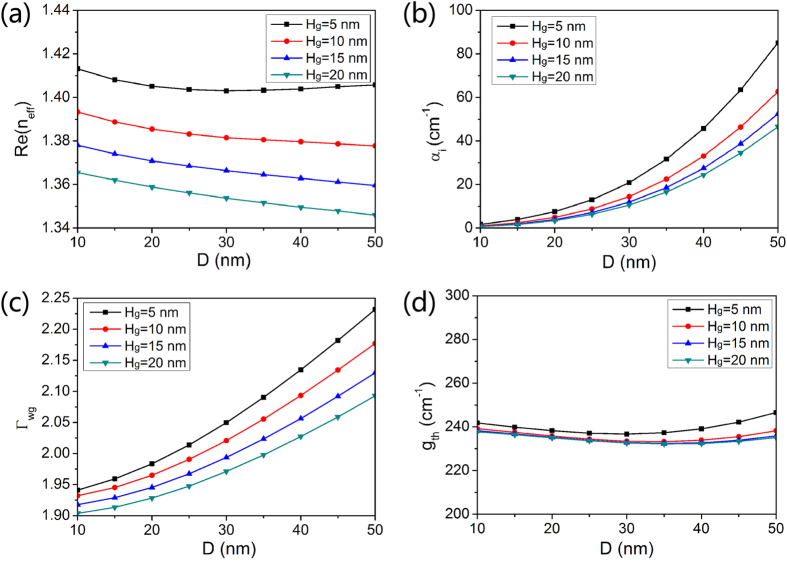
Guiding and cavity properties of the nanolaser. Dependences of (**a**) the real part of the effective index, (**b**) modal loss, (**c**) modal confinement factor and (**d**) threshold gain on nanowire dimeter *D* and low-index strip height *H*_*g*_.

To investigate the cavity characteristics of the proposed nanolaser, dependences of the real part of effective index, modal loss, modal confinement factor and threshold gain on *D* and *H*_*g*_ are calculated and presented in Fig. 4(a–d). As shown in Fig. 4(a), the real part of the effective indices Re(*n*_eff_) decrease with the increasing diameter. As the low-index height *H*_*g*_ decreases, the enhanced coupling strength between the modes of Ag nanowire and dielectric structure increases Re(*n*_eff_). The modal loss per unit length α_*i*_ and modal confinement factor Γ_*wg*_ are two significant factors of the optical cavity relevant to lasing. The modal confinement factor Γ_wg_ is an indicator of how well the mode overlaps with the gain medium, which is defined as the ratio between the modal gain and material gain in the active region^25^,^26^,^27^. The equations in the calculation are narrated in the last methods section. As shown in Fig. 4(b), when *D* is small, α_*i*_ is quite low, which is attributed to the small metal dissipation. Conversely, the narrow low-index gap localizes electromagnetic energy around the Ag nanowire, giving rise to large metal dissipation and α_*i*_. The modal confinement factor as functions of *D* and *H*_*g*_ are shown in Fig. 4(c). A minimum Γ_*wg*_ of 1.90 occurs at *D* = 10 nm and *H*_*g*_ = 20 nm. Γ_*wg*_ exhibits positive correlations with *D* and negative correlations with *H*_*g*_. The maximum Γ_*wg*_ of 2.23 occurs at *D* = 50 nm and *H*_*g*_ = 5 nm. This indicates that the field of the hybrid plasmonic mode is highly confined in the F-P cavity and overlaps well with the active region.

Lasing threshold is the lowest excitation level at which laser output is dominated by stimulated emission rather than spontaneous emission. The threshold gain g_th_, which describes the required gain per unit length for lasing, is defined as 
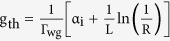
, where *R* denotes the geometric mean of the reflectivity of the Ag reflecting mirrors and *L* is the length of the F-P cavity^28^. Threshold gain is dominated by mirror loss rather than modal loss, if the nanocavity has a cavity length as short as a few micrometers. So, thickening Ag reflectors is an efficient way to enhance the reflectivity and provides a solution to problem of the high threshold gain. The two Ag-coated end facets of the nanolaser exhibit reflectivity higher than 95% when the thickness of the Ag coating is >50 nm, which is an efficient way to reduce the threshold gain for lasing^29^. Hence, the thickness and the reflectivity *R* of the Ag coating are assumed to be 50 nm and 95% in the following calculations. The length of the nanolaser *L* is fixed at one effective wavelength to ensure that standing wave can stably oscillate in the cavity and all three dimensions of the nanolaser are smaller than the free-space wavelength with the sufficient gain provided by the semiconductor gain medium. We depict dependence of threshold gain g_*th*_ on *D* and *H*_*g*_ in Fig. 4(d). It is obvious that the threshold gain g_*th*_ keeps stable and has a little variation around 240 cm^−1^ for different *D* and *H*_*g*_. Low threshold gain means that laser could easily lase at low gain level. The threshold gain and cross-section area of the nanolaser in this paper are comparable to semiconductor nanowire lasers, but the cavity length is quite smaller than the length of nanowires^10^,^11^,^30^. All the three dimensions of the proposed nanolaser are at the subwavelength scale which is advantageous in compactness and integration. By increasing the cavity length of the nanolaser, g_*th*_ can be decreased below 50 cm^−1^, which will be elaborated in the last section.

**Figure 5:**
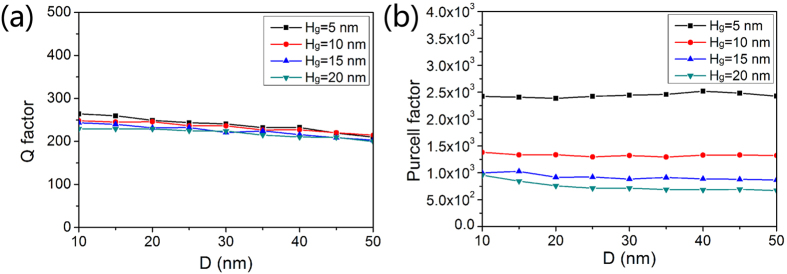
Lasing properties of the nanolaser. (**a**) Quality factor Q and (**b**) Purcell factor as functions of *D* and *H*_*g*_.

Quality factor Q of a cavity mode is indicative of how long the stored energy of that mode remains in the cavity when interband transitions are absent, which is related to the photon lifetime τ_p_ enters the rate equation via the resonance frequency ω of the mode. As the equation of quality factor mentioned in ref. 31, the dispersion resulting in the obvious group velocity along with the quality factor Q is much more significant in the plasmonic cavity with a small core than in the conventional semiconductor dielectric cavity. High quality factor indicates a low rate of energy loss relative to the stored energy of the cavity and the oscillations die out slowly. So the device can lase at a lower threshold and hence pump power could be reduced. We depict Q as functions of *D* and *H*_*g*_ in Fig. 5(a). Q factors keep stable with small fluctuations around 250 for different *D* and *H*_*g*_.

On the other hand, spontaneous emission rate in the nanolaser partly depends on environment of a light source. According to Fermi’s golden role, the spontaneous emission rate of an emitter is proportional to the local density of optical states (LDOS)^32^. In an environment that structure is at the scale of the wavelength, the LDOS can be spatially controlled^33^. As a result, the LDOS of an emitter can be locally increased together with the rate of spontaneous emission or decreased by the subwavelength microcavity, which is called the Purcell effect^34^. The nanolocalized electromagnetic field of the symmetric hybrid plasmonic mode in the nanolaser can decrease the lasing threshold by enhancing the spontaneous emission rate via the Purcell effect. In the proposed nanolaser, the Ag nanowire is separated from semiconductor gain material by a nanoscale dielectric gap, enabling part of electromagnetic energy confined in the nanoscale dielectric gap. The asymmetric plasmonic structure modifies the dielectric environment and constructs a nanocavity at the subwavelength scale, in which the nanolocalized electromagnetic field of hybrid plasmonic modes enables an ultrasmall volume and coupling between an exciton and a microcavity mode^35^. As a result, the rate of spontaneous emission is enhanced via the Purcell effect. Moreover, a large LDOS can enhance not only the rate of spontaneous emission, but also stimulated emission processes in the lasing action^36^. Lasing action could be easier achieved because the nanolocalized electromagnetic field of the hybrid plasmonic mode not only makes the excitons in the nanolaser diffuse rapidly towards areas of faster recombination improving the overlap between material gain and plasmonic mode but also stimulates excited-state particles to transfer energy into plasmons of the same frequency, phase and polarization. According to the results of theoretical calculations in ref. 36, only the population inversion in the subwavelength vicinity of the SiO_2_ gap can effectively participate in the lasing action.

The enhancement of spontaneous emission rate induced by the Purcell effect can be quantified by the Purcell factor. A large *Q* and a small modal volume *V*_eff_ are desired for strong Purcell effect and large spontaneous emission rates. One advantage of the proposed nanolaser is the nanoscale optical modal size due to the high confinement of the plasmonic mode. So this nanostructure of the cavity can significantly increase the Purcell factor. The Purcell factor *F*_*p*_ as functions of *D* and *H*_*g*_ are shown in Fig. 5(b). *F*_*p*_ increases with *H*_*g*_, but remains stable with increasing *D*. Smaller *H*_*g*_ enhances the coupling between plasmonic mode and dielectric mode, resulting in smaller effective modal volume *V*_eff_. Therefore, the Purcell factors for *H*_*g*_ = 5 nm are the largest ones and the maximum Purcell factor can be as large as 2518 when *H*_*g*_ = 5 nm and *D* = 40 nm. Even when *H*_*g*_ = 20 nm, the Purcell factors around 750 are still very high compared to some other microcavities^37^,^38^,^39^.

**Figure 6:**
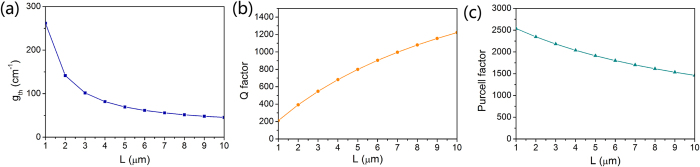
Lasing properties with varying cavity length. Dependences of (**a**) g_*th*_, (**b**) quality factor *Q* and (**c**) Purcell factor on *L*.

As the equations shown in the methods section, the length of the cavity *L* is a significant parameter impacting on the threshold gain, quality factor and Purcell factor. Dependences of threshold gain g_*th*_, quality factor *Q* and Purcell factor on the length of the cavity *L* are presented in Fig. 6 to get insight into the influence of *L* on the lasing characteristics of the nanolaser. *D* and *H*_*g*_ are fixed at 40 nm and 5 nm, respectively. Figure 6(a) reveals that g_*th*_ decreases descending trend as *L* increases. When *L* is more than 7 μm, the threshold gain could be as low as ~50 cm^−1^. It is obvious that a longer nanolaser realizes larger gain volume and longer roundtrip path of lightwave in the gain medium, which leads to lower threshold gain. As *Q* is inversely proportional to g_*th*_, a longer cavity length indicates that the stored energy of that mode remains in the cavity long is longer, which is related to the photon lifetime τ_p_ enters the rate equation via the resonance frequency of the mode. Hence, *Q* increases as the length *L* increases, as shown in Fig. 6(b). In Fig. 6(c), the Purcell factor decreases from 2539 to 1462 with increasing *L*. Therefore, *L* = one effective wavelength could make the nanolaser satisfy all the three dimensions below the free-space wavelength. In the meantime, the Purcell factor can be as large as 2518. Whereas, the cavity length ranges from 5 to 7 μm could simultaneously realize the low threshold gain, high quality factor and Purcell factor, which makes lasing easier with moderate laser volume.

In addition, the effect of fabrication errors of Ag nanowire diameter and low-index dielectric gap height on the lasing characteristics can be neglected. An important concern would be that slight slopes in the sidewalls might be harmful factors to lasing of the nanolaser. However, this nanolaser is also quite tolerant to slight sidewall slopes since the majority of electromagnetic energy is tightly confined around the Ag nanowire in the low-index region. Based on the above, the nanolaser can behave single-mode lasing emission and the lasing characteristics keep stable and good performance despite the structure parameters changes, which is highly significant in the industrial production.

## Discussion

We have proposed a nanolaser based on an asymmetric hybrid plasmonic structure, taking full advantage of its low modal loss and strong nanoscale modal confinement. Its end facets are Ag-coated to provide high reflectivity and form F-P cavity. All three dimensions of the nanolaser are smaller than the free-space wavelength. The lasing characteristics of the proposed nanolaser have been numerically analyzed using FEM at a wavelength of 1550 nm. The nanolaser has a threshold gain of 240 cm^−1^ when its cavity length is one effective wavelength. In the meantime, an ultrahigh Purcell factor of 2518 are predicted due to the nanocavity constructed by the asymmetric plasmonic structure, which could provide low threshold and enhanced rates spontaneous and stimulated emission via the Purcell effect to make the lasing easier. In addition, the nanolaser exhibits quite robust characteristics with respect to certain fabrication imperfections, such as the deviations in the diameter of Ag nanowire and slight changes in the sidewall slopes. So the proposed low-threshold subwavelength nanolaser with ultrahigh Purcell factor is promising in applications of on-chip interconnects, photonic integrated circuits, optical storage and optical signal process.

## Methods

### Numerical calculation

The modal and lasing characteristics of the proposed nanolaser are theoretically investigated using the finite element method (FEM) with the scattering boundary condition, which is a commonly employed approach to mimic the necessary open boundary. The modal field distributions of the eigenmodes in the nanolaser are directly obtained by mode analyses. The characteristics of the hybrid plasmonic mode are calculated by the complex propagating constant with *β* + i*α*. The real part of the modal effective index is calculated by *n*_*eff*_ = Re(*N*_*eff*_) = *β*/*k*_*0*_, where *k*_*0*_ is the vacuum wavevector. The effective mode area is calculated using^16^:


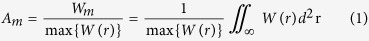


where *W*_*m*_ is the total mode energy and *W*(*r*) is the energy density (per unit length flowed along the direction of propagation). For dispersive and lossy materials, the *W*(*r*) inside can be calculated using the following equation:


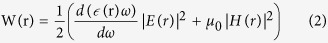


### Guiding properties

The modal loss per unit length α_*i*_ can be obtained from the imaginary part of modal propagation constant *k*_*z*_ as α_*i*_ = 2Im[*k*_*z*_]. The modal confinement factor Γ_*wg*_ is defined as the ratio between the modal gain and material gain in the active region^25^,^26^,^27^:


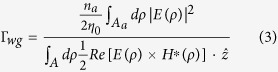


where *η*_0_ is the intrinsic impedance; *n*_*a*_ is the index of the active region; *A*_*a*_ is the cross section of the active region; *A* is the whole cross section ideally extending to the infinity; *E* and *H* are the complex electric and magnetic fields of the guided modes.

### Lasing Properties

For a F-P cavity, the quality factor *Q* is defined as^31^:


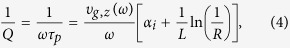


where 
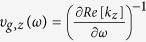
 is the group velocity of the guided mode. Purcell factor which is defined as^34^:


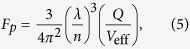


where (λ/n) is the wavelength within the material and *V*_eff_ is the effective modal volume of the cavity.

## Additional Information

**How to cite this article**: Wei, W. *et al*. Ultrahigh Purcell factor in low-threshold nanolaser based on asymmetric hybrid plasmonic cavity. *Sci. Rep.* **6**, 33063; doi: 10.1038/srep33063 (2016).

## Figures and Tables

**Figure 1:**
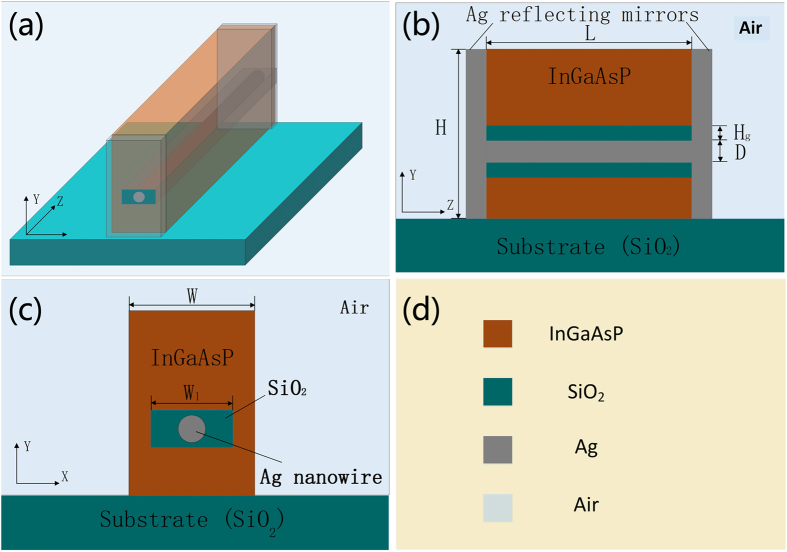
Schematic diagram of the nanolaser. (**a**) 3D top view, (**b**) Y-Z cross-section view, (**c**) X-Y cross-section view, and (**d**) annotation of the materials.
